# Advancing EEG prediction with deep learning and uncertainty estimation

**DOI:** 10.1186/s40708-024-00239-6

**Published:** 2024-10-26

**Authors:** Mats Tveter, Thomas Tveitstøl, Christoffer Hatlestad-Hall, Ana S. Pérez T., Erik Taubøll, Anis Yazidi, Hugo L. Hammer, Ira R. J. Hebold Haraldsen

**Affiliations:** 1 Department of Neurology, Oslo University Hospital, Oslo, Norway; 2 Institute of Clinical Medicine, Faculty of Medicine, University of Oslo, Oslo, Norway; 3 Department of Computer Science, Oslo Metropolitan University, Oslo, Norway; 4 Department of Holistic Systems, SimulaMet, Oslo, Norway

**Keywords:** Deep learning, EEG, Artificial intelligence, Ensembles, Uncertainty, Machine learning

## Abstract

Deep Learning (DL) has the potential to enhance patient outcomes in healthcare by implementing proficient systems for disease detection and diagnosis. However, the complexity and lack of interpretability impede their widespread adoption in critical high-stakes predictions in healthcare. Incorporating uncertainty estimations in DL systems can increase trustworthiness, providing valuable insights into the model’s confidence and improving the explanation of predictions. Additionally, introducing explainability measures, recognized and embraced by healthcare experts, can help address this challenge. In this study, we investigate DL models’ ability to predict sex directly from electroencephalography (EEG) data. While sex prediction have limited direct clinical application, its binary nature makes it a valuable benchmark for optimizing deep learning techniques in EEG data analysis. Furthermore, we explore the use of DL ensembles to improve performance over single models and as an approach to increase interpretability and performance through uncertainty estimation. Lastly, we use a data-driven approach to evaluate the relationship between frequency bands and sex prediction, offering insights into their relative importance. InceptionNetwork, a single DL model, achieved 90.7% accuracy and an AUC of 0.947, and the best-performing ensemble, combining variations of InceptionNetwork and EEGNet, achieved 91.1% accuracy in predicting sex from EEG data using five-fold cross-validation. Uncertainty estimation through deep ensembles led to increased prediction performance, and the models were able to classify sex in all frequency bands, indicating sex-specific features across all bands.

## Introduction

Recent methodological advancements in deep learning (DL) have made it particularly promising for applications in healthcare [1–3]. DL, a specialized sub-branch of artificial intelligence (AI) and machine learning (ML) [4], is designed to construct complex models with multilayered architectures that excel in feature extraction from high-dimensional, complex datasets [5]. Unlike traditional techniques that rely on manually crafted features, DL uses data-driven optimization to automatically extract key features, uncovering data relationships that might otherwise be missed. This has positioned DL as a powerful tool for tackling complex challenges, particularly in fields with rich, multidimensional data.

An emerging area within DL is uncertainty modeling, which is crucial for applications in safety-critical domains such as healthcare [6]. DL models inherently face challenges with variability and ambiguity in data, making it essential to quantify uncertainty to improve model interpretability and trustworthiness. Uncertainty in DL is typically categorized into aleatoric uncertainty, which arises from inherent data noise, and epistemic uncertainty, which stems from the model’s lack of knowledge about the data [7]. Estimating uncertainty in DL models is generally achieved through Bayesian approaches or ensemble methods. Ensemble methods, which aggregate the predictions of multiple models, have shown strong performance across various domains by addressing the weaknesses of individual models [8]. The distribution of predictions within an ensemble provides a direct measure of the model’s uncertainty, offering a quantifiable assessment of confidence in its outputs. Analyzing the variability among these predictions can significantly enhance the model’s interpretability and reliability. Moreover, beyond uncertainty estimation, DL ensembles may offer significant performance advantages. Single DL models often encounter challenges such as local minima, plateaus, and saddle points in the optimization landscape, which can hinder training and reduce overall model accuracy [4, 9]. Ensemble methods mitigate these challenges by combining models that explore different regions of the optimization space. This diversity allows ensembles to generate more robust and accurate predictions, as they can effectively navigate the complex landscape that a single model might struggle with. Addressing the inherent uncertainties in healthcare-related DL models could aid in evaluating a prediction’s reliability. By addressing these inherent uncertainties, particularly in healthcare-related DL models, ensembles can help evaluate the reliability of predictions. This uncertainty identification is crucial for providing medical professionals with the insights needed for better-informed decision-making, fostering a collaborative process between AI systems and healthcare providers, ultimately leading to improved patient outcomes.

Electroencephalography (EEG) is a powerful and widely used tool in neuroscience and clinical practice due to its non-invasive nature, cost-effectiveness, and temporal resolution. The clinical utility of EEG is significant, with its cost-efficiency stemming from expenses largely limited to the clinician’s time, making it far more affordable than other imaging techniques like MRI. Its non-invasive nature reduces patient risk and discomfort, and its portability enables use in diverse settings, including resource-limited regions, highlighting its critical role in neurological diagnosis and monitoring. EEG measures the brain’s electrical activity using scalp electrodes that record a summation of neuronal activity, with the proximity and orientation of the signal generators as important mediators. While EEG provides high temporal resolution, capable of capturing potential fluctuations on a millisecond scale, it suffers from poor spatial resolution due to i) the smearing effect of the head as a volume conductor and ii) relatively poor signal-to-noise ratio due to intrinsic and extrinsic artifacts [10]. EEG systems vary in the number of electrodes, from 19 in clinical applications to 256 and beyond in research [11]. The complexity and multidimensionality of EEG necessitates more sophisticated analytical techniques to fully harness the rich information contained within these signals. Traditional ML approaches often rely on human-engineered features, which account for approximately 50% of EEG-based ML/DL publications [12]. However, this method carries the risk of overlooking critical patterns embedded in the data. In contrast, DL models, particularly those designed for time-series data like EEGNet [13] and InceptionTime [14], offer the capability to automatically detect subtle and complex patterns, including the temporal dependencies of EEG data, that may be missed by traditional feature engineering.

In this study, we focus on the prediction of sex from EEG data. Although, the clinical utility of predicting sex is limited, this task was selected because sex is a binary and definitive label, which contrasts with the inherent variability and uncertainty associated with other clinical diagnoses. The binary nature of sex as a label offers a clear and unambiguous target, making it an ideal benchmark for evaluating the performance of DL models on EEG data. Given that DL models require large datasets to achieve robust generalization, and many EEG datasets already include sex as a label, this prediction task is both practical and accessible for testing and validating model performance. The findings from this study will serve as a foundation for future research, where these models will be applied to more complex prediction tasks in EEG.

While sex prediction provides a clear and binary label, EEG prediction tasks are inherently complex and face multiple challenges. EEG signals are affected by noise, leading to a low signal-to-noise ratio, and despite cleaning efforts, some noise typically remains, making it challenging to extract reliable neural activity patterns. The high dimensionality of the data adds complexity, and the variability among subjects, combined with the non-stationary nature of EEG, complicates generalization across individuals [12]. Lastly, EEG signals rarely provide clear patterns for accurate prediction-for instance, it’s not possible to visually determine whether an EEG belongs to a male or female. This contrasts with prediction tasks using X-rays or MRI, where relevant features are often visible to medical professionals, and AI is used to speed up evaluation. In the case of EEG, the challenge is not merely accelerating analysis but whether models can reveal new insights.

This study makes several key contributions. First, while sex prediction itself may have limited clinical significance, we propose a baseline for prediction accuracy using a large EEG dataset, which serves as a valuable reference for future research. Second, we challenge the prevailing assumption that the beta band is the optimal predictor of sex in EEG data by demonstrating that strong predictive performance can be achieved across all frequency bands. This finding offers a new perspective on EEG-based sex prediction. Finally, our exploration of uncertainty in deep learning ensembles provides insights into how uncertainty can be used to improve model performance. This study is part of the European AI-Mind [15] Research & Innovation action (No 964220), dedicated to developing AI-based decision tools for early dementia risk detection.

## Methods

### Data and preprocessing

#### Dataset

The Child Mind Institute [16] offers an open-source dataset featuring various medical imaging data modalities. The population consists of children aged between 5 and 21 years, with varying pathologies, not individually detailed in the dataset. This work focuses on resting state EEG data only. The dataset was balanced for sex by removing overrepresented subjects. After discarding subjects with insufficient data, the balanced dataset contained 1780 subjects. This dataset was selected due to its large size, which is crucial for deep learning, as it allows for more effective training and validation, reducing the risk of overfitting and enhancing the generalization of the models. Additionally, the chosen age range covers critical developmental stages, including childhood and adolescence, which are characterized by significant physiological and cognitive changes. These changes introduce variability in EEG signals, providing a robust test for our models’ adaptability and performance.

#### EEG preprocessing and data preparation

Raw EEG signals were preprocessed using an automated MATLAB cleaning pipeline, using EEGLAB toolbox [17]. Channels with low-quality data were eliminated through an iterative process, where signals with an amplitude standard deviation (SD) greater than 75 $\mu$V or no amplitude variation were excluded. EEG files were rejected if more than 30% of channels were excluded. The line artifacts were removed using Zapline software [18], and a band-pass filter from 1 to 45 Hz was applied to the signals. To maintain data dimension consistency, excluded channels were substituted with interpolated signals. The pipeline is available here.

The cleaned EEG data consisted of 129 channels with a 500 Hz sampling rate and approximately six minutes of EEG per subject. The initial 30 s of each subject’s EEG were discarded. Depending on the algorithm development’s training, validation, or testing split, 1–2 min of the EEG was extracted and divided into epochs of 2 s. Given the high correlation between successive EEG epochs, every other epoch was removed from the 120-second training set, enhancing the diversity. The 60-second validation and 80-second test set was segmented into successive epochs and analyzed.

#### Data split

The model’s performance was evaluated using a five-fold cross-validation scheme to ensure robustness across multiple iterations. The splits were generated once by randomly assigning the male and female subjects to the five folds, resulting in 356 subjects per fold, with equal distribution of males and females within each fold. This guaranteed consistent subject use across all experiments, maintaining evaluation process uniformity. The cross-validation scheme was iterative. It involved training the model on three out of five folds, validation on one, and testing on the remaining fold. This process is repeated iteratively to ensure a comprehensive evaluation of the model’s performance across different subsets of the data. The cross-validation scheme robustly assessed the model’s generalization capabilities by rotating the folds and systematically training, validating, and testing on different subsets. This methodology is equal to a dataset split of 60% (1070 subjects) for training, 20% (356 subjects) for validation, and 20% (356 subjects) for testing. Folds were independent; no subject appeared in more than one test set across the five runs.

### Hyperparameters and evaluation

#### Hyperparameters

The model was trained for 50 iterations, with the validation set used for continuous performance monitoring. Metrics derived from the validation set guided the decision to stop training, employing techniques such as early stopping and learning-rate scheduler. Early stopping was implemented to prevent overfitting while decreasing the learning rate during training ensures more efficient convergence towards an optimal solution. The best-performing model, determined by the validation set’s loss, was saved to avoid overfitting and evaluated against the final model obtained after training. Only the best-performing model was retained for further use. In this study, early stopping was set with patience of 15 iterations, halting training if the validation set’s loss did not decrease. The adaptive learning rate used a patience of 5 iterations, starting with an initial value of 0.005. The Glorot uniform initialization method [19] was primarily used to initialize weights, except in cases where a random normal or random uniform distribution was employed for an ensemble of models. The training was conducted on a computer with an NVIDIA GeForce RTX 3060 12GB GPU.

#### Test set evaluation

The performance on the test set was evaluated using three primary metrics: per-subject accuracy, per-epoch accuracy, and AUC. Per-subject accuracy was derived through a majority voting scheme applied to the test set. Here, each of the 40 2-second EEG epochs was individually predicted as male or female. The majority of the prediction determines the per-subject sex prediction. The per-epoch accuracy evaluated each 2-second epoch as an individual data point without consolidating predictions per subject. This approach provided a detailed performance view of the model. AUC measures a binary classification model’s performance. It quantifies the classifier’s ability to differentiate between positive and negative classes. An AUC value of 1 signifies perfect distinction, while 0.5 indicates no better performance than guessing.

A majority voting strategy was used and applied in two distinct methodologies for the ensemble evaluation. The first is the “Ensemble Subject” method. Each model in the ensemble independently performed a majority voting procedure on a subject, and the most frequently predicted sex across all ensembles was then identified as the final prediction for that subject. The second method is the “Ensemble Epoch”. Each model predicted each epoch, and the average prediction per epoch was calculated across all ensemble models. The majority prediction within a subject was derived from these averaged predictions.

### Models

The DL model was developed using Keras [20] and designed to receive EEG time series as input. Data processing was managed by numpy [21] and mne-python [22].

#### Single models

Two deep learning models, InceptionNetwork [14] and EEGNet [13], were selected for sex prediction based on their proven effectiveness in relevant domains, with their suitability for this task also confirmed through experimentation. InceptionNetwork, a robust model for time-series classification, was chosen due to its strong performance across a wide range of time-series tasks. InceptionNetwork, utilizes inception modules which combines different length kernels with pooling operations followed by merging them. The architecture consists of a series of these modules, enhanced by skip connections that facilitate information flow across the layers. A depth of 4 was identified through experimentation as the most effective setting, underlining the importance of fine-tuning models for specific tasks. EEGNet, a convolutional neural network originally developed for EEG-based brain-computer interface tasks, was chosen for this study due to its prior success in handling EEG data. Its proven ability to extract relevant features from EEG signals made it a strong candidate, and it was adapted to address the specific challenge of sex prediction in this context. Unique in its use of depth-wise convolutions and separable convolutional layers, EEGNet allows for efficient and expressive feature extraction. Both InceptionNetwork and EEGNet architecture underwent modifications to create various ensemble models (these changes can be found in the supplied code). These alterations, facilitated ensemble techniques such as Adapted Dropout Strategy and depth-ensembles. Most changes were related to depth in cases that were not specific ensemble types.

#### Deep learning ensembles

DL ensembles can be created using several strategies, each with benefits and limitations. The adopted method in this study entails the combination of different models which differ in their architecture, weight initialization, or depths. The objective is to leverage an ensemble of models that, despite sharing a common task, examine the data from different perspectives due to their unique characteristics. The general benefit is the merged predictions from various models, leading to a more balanced and robust final prediction. A depth ensemble is one such example. Changing the depths of the models allows for a more diverse set of learned features. As a model’s depth increases, so does the abstraction level of the features it captures. As a result, a depth ensemble becomes more robust and provides a more thorough understanding of the underlying data structures. The major limitation of deep ensembles is that training multiple models is computationally expensive, except in the case of Monte Carlo Dropout.

##### Adapted dropout strategy

Monte Carlo Dropout (MCD) [23] introduces randomness in the activation of artificial neurons in the neural network during the model’s testing phase. By deactivating a percentage of artificial neurons with a probability *P*, an ensemble of models with slightly different architectures is generated based on which neurons are activated or deactivated. The number of forward passes was set to 50, drawing on insights from previous studies [6, 23–25], to obtain a reliable estimate of both the predictive accuracy and the uncertainty associated with the ensemble’s output. The ensemble’s output was then determined by calculating the mean of these forward passes. The varied artificial neuron settings yield an ensemble of models, each giving a slightly different data interpretation.

To approximate a Bayesian probabilistic deep Gaussian process the incorporation of a dropout layer before each weight layer is recommended [23]. In our methodology, we deviated from this recommendation by introducing a modification: the addition of a dense layer with 32 neurons, followed by a dropout layer, to the base models, InceptionNetwork and EEGNet. This Adapted Dropout Strategy (ADS) simplifies the ensemble generation process, enabling the exploration of performance enhancements and straightforward uncertainty estimations, without fully committing to a Bayesian network approach. The rationale behind this choice stems from the original models’ strong performance, suggesting that an ensemble with minor variations could offer additional benefits. The selected dropout rate was set at 0.5, consistent with the classification task in [23] MC Dropout study. A significant advantage of this technique is that it necessitates training only a single model, making it less computationally intensive than other ensemble strategies.

##### Weight randomization

In this ensemble, the starting weights utilized a random uniform or random normal distribution as the kernel initializer. This strategy ensures that each model in the ensemble begins its training with a unique set of initial weights. Consequently, even though all models are trained on the same dataset and share the same architectures, the disparity in the starting position leads to slightly different learning paths during the training process. The finalized trained models exhibit minor variations in their internal representation and, by training the model 5 times, resulting in an ensemble of different models for InceptionNetwork and EEGNet.

##### Depth ensemble

In the original InceptionNetwork model, depth is a hyperparameter that can be manipulated to adjust the model’s complexity. This study created a range of models with varying depths, specifically (2, 4, 6, 8, 10), to form a depth ensemble.

##### Model ensemble

This ensemble was constructed using previously trained models, consisting of the original InceptionNetwork and EEGNet, combined with models with depth variations and ADS versions.

##### Frequency ensemble

The frequency-focused ensemble was constructed by training instances of the same model on distinct EEG signal frequency bands in the time domain, specifically delta (0.5–4Hz), theta (4–8Hz), alpha (8–12Hz), low-beta (12–20Hz), high-beta (20–30Hz), and gamma (30–45Hz). This method differs from traditional ensemble bagging, which typically partitions data at the dataset level, not based on signal attributes like frequency bands. The strategy enables each model to gain and apply insights from its assigned frequency band, specializing in those unique characteristics. The three most effective models, determined by validation set performance, were selected to compose the final ensemble. First, this approach created an ensemble of models, each specializing in a distinct EEG frequency range, evaluated with the unfiltered EEG signal. Second, it laid the groundwork for investigating the role of various frequency bands in distinguishing biological sex, based on the predictive performance of models trained and tested on these bands using EEG data.

### Uncertainty - choosing the most certain parts of an EEG

Each subject’s data was divided into multiple epochs, with each epoch being predicted multiple times-specifically, *n* times, where *n* represents the number of models in the ensemble. To assess the uncertainty of the model, a simple uncertainty measure was calculated based on the predictions of the ensemble for each epoch. This measure aimed to evaluate the degree of consensus or disagreement among the models in the ensemble regarding the prediction of a given epoch.

**Fig. 1:**
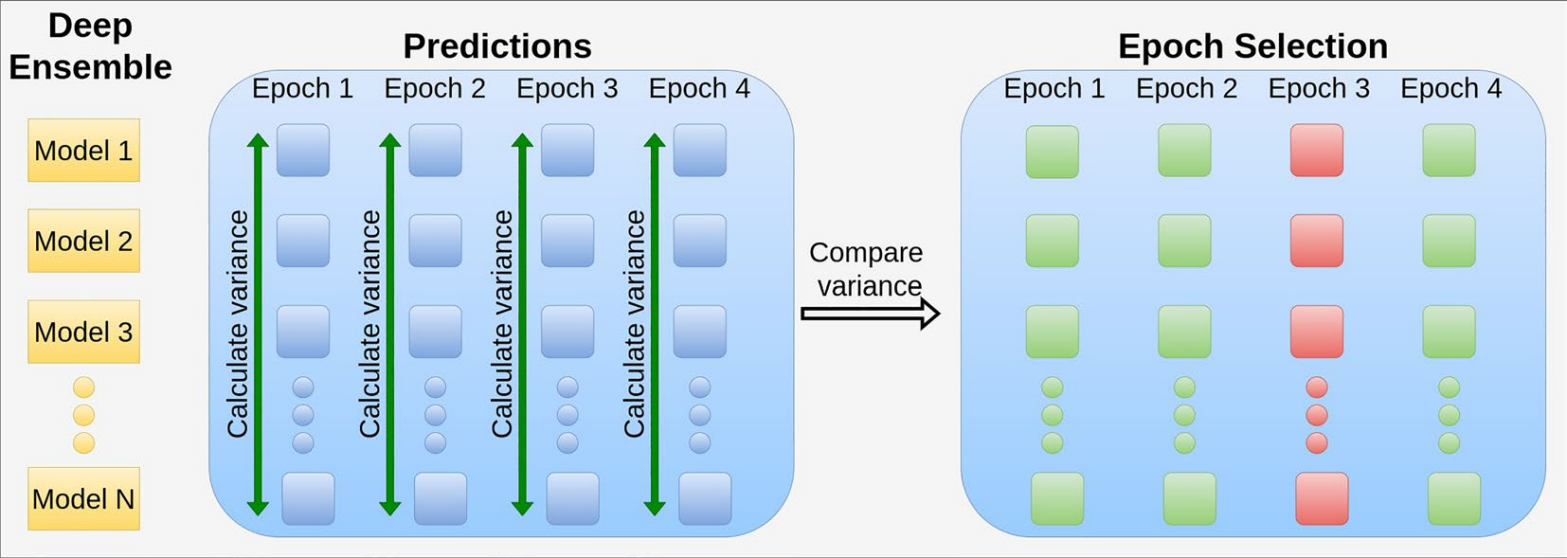
*N* deep ensembles predict each epoch, with the variance calculated across each epoch over the ensemble predictions. Epochs exhibiting the greatest variance are discarded. The percentage *p* value increases, and the epochs are evaluated with a continuously decreasing number of epochs. The figure includes four example epochs, each predicted by the ensemble models and variance is computed. The epoch with the highest uncertainty (Epoch 3) is subsequently eliminated from the pool. This process demonstrates our method for handling and reducing prediction uncertainty in EEG analysis

The uncertainty was quantified using a basic variance metric on the predicted probabilities across all models in the ensemble for each epoch. This variance indicated the quality and reliability of the predictions, with a higher variance suggesting greater disagreement among the models. Following this, the percentage *p* of epochs with the highest uncertainty of variance was identified (see Fig. 1 for a visual representation of the method). To evaluate the impact of uncertain predictions on overall model performance, we systematically removed the most uncertain epochs, starting with the top 10% of epochs with the highest variance. The remaining epochs were then re-evaluated to observe any changes in model performance. This process was repeated by progressively removing 25%, 50%, 75%, and finally 90% of the most uncertain epochs. The performance across the test set was averaged to determine whether excluding uncertain epochs led to a general improvement in model accuracy.

## Results

### Single model sex prediction

**Table 1:** Single Model Performance: Comparing performance between InceptionNetwork and EEGNet in sex prediction from EEG data

Model name	Per epoch	Per subject	AUC
EEGNet	88.3 ± 1.5	90.5 ± 1.6	0.938 ± 0.01
InceptionN.	88.0 ± 1.6	90.7 ± 2.4	0.947 ± 0.01

Mean accuracy accompanied by a 95% confidence interval. It also provides a detailed analysis of per-subject and per-epoch accuracy, as well as the area under the curve (AUC) to assess the overall performance of each model

InceptionNetwork and EEGNet demonstrated superior capabilities in predicting sex directly from EEG, achieving mean performance exceeding 90% across a 5-fold cross-validation, as seen in Table 1. Both models performed well with respect to both per epoch and per subject, as well as the AUC. These observations suggest that both models are robust in generalization and discriminate ability for the given problem. Notably, the differences in results were not statistically significant, which contributed to our decision to include variants of both models in the ensembles.

### Deep ensembles

**Table 2:** Ensemble Model Performance: Performance of the DL ensembles in predicting sex from EEG data: Each ensemble was run using five-fold cross-validation, with five models in each ensemble, except for ADS (50) and the model ensemble, which utilized already trained models

Model name	Ensemble epoch	Ensemble subject
Inception ADS	90.4 ± 1.0	90.4 ± 1.0
EEGNet ADS	86.2 ± 3.4	86.2 ± 3.4
Inception depth ensemble	90.6 ± 1.7	90.9 ± 1.8
Inception weights (normal)	90.8 ± 1.1	91.0 ± 1.1
Inception weights (uniform)	90.3 ± 1.3	90.6 ± 1.4
EEGNet weights (normal)	90.6 ± 1.2	90.7 ± 1.3
EEGNet weights (uniform)	90.2 ± 1.5	90.6 ± 1.5
Model ensemble	91.1 ± 1.3	91.1 ± 1.2
Freq. ensemble (3 bands)	86.6 ± 6.5	86.7 ± 7.3

The table displays the mean accuracy paired with a 95% confidence interval, providing a comprehensive view of each ensemble’s performance in sex prediction tasks

The performance of various ensemble methods is summarized in Table 2, with all types achieving an accuracy rate of approximately 90%. No significant differences were observed among the ensemble types or between the epoch-level and subject-level approaches. Notably, wider confidence intervals for both EEGNet ADS and the frequency ensemble suggested greater variability across runs and folds. This variability was especially pronounced in the frequency ensemble, where model performance varied significantly across folds. The performances of all ensembles were comparable to those of the single models. The confidence interval provides insight into the statistical disparity between the performance of ensembles 2 and the performance of the single models 1. The results reveal no statistically significant improvement, with no ensemble achieving performance so superior that their confidence intervals do not overlap with those of the single models.

### Frequency-based explainability

**Fig. 2:**
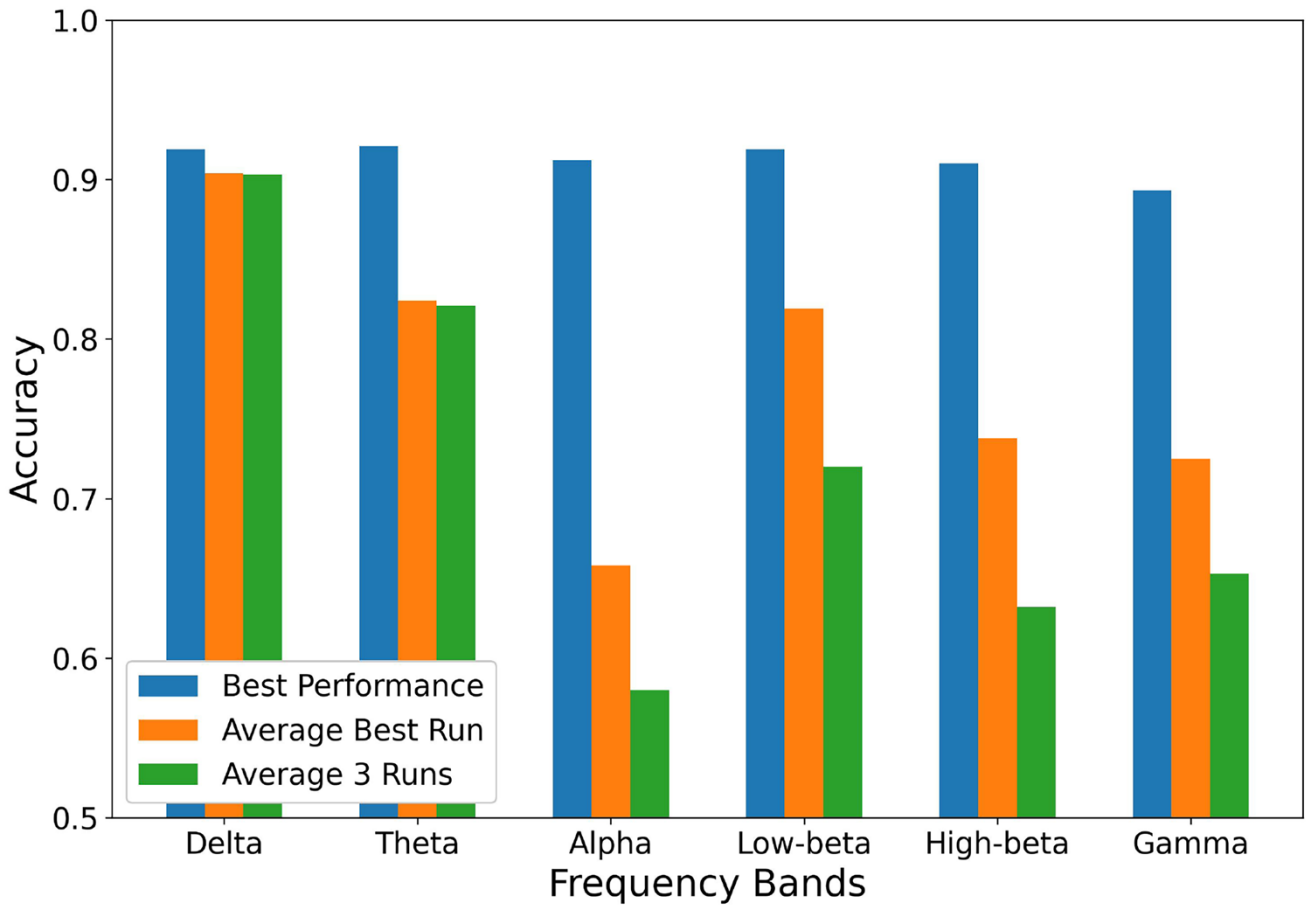
Performance of the InceptionNetwork model when individual frequency bands are used as input. Performance is categorized into three sections: (1) Best performance across all individual folds, (2) Best average performance across a run of 5-folds, and (3) Average performance across three separate runs. The performance results are derived from the test set, utilizing majority voting for final predictions. This illustration provides insight into the model’s adaptability and efficiency across varying frequency bands

Figure 2 presents performance metrics for various EEG frequency bands. The blue bars, indicating the peak performance within a single fold from all k-folds over three runs, show minimal variation among the bands, with each hovering around a 90% performance level. A more pronounced difference in performance emerges when examining the orange bars (the best 5-fold run from three attempts) versus the green bars (the average performance over three 5-fold runs). Specifically, the delta, theta, and low-beta bands outperform the others, evidenced by their superior scores in both the orange and green metrics.

### Uncertainty - epoch rejecting performance

**Fig. 3:**
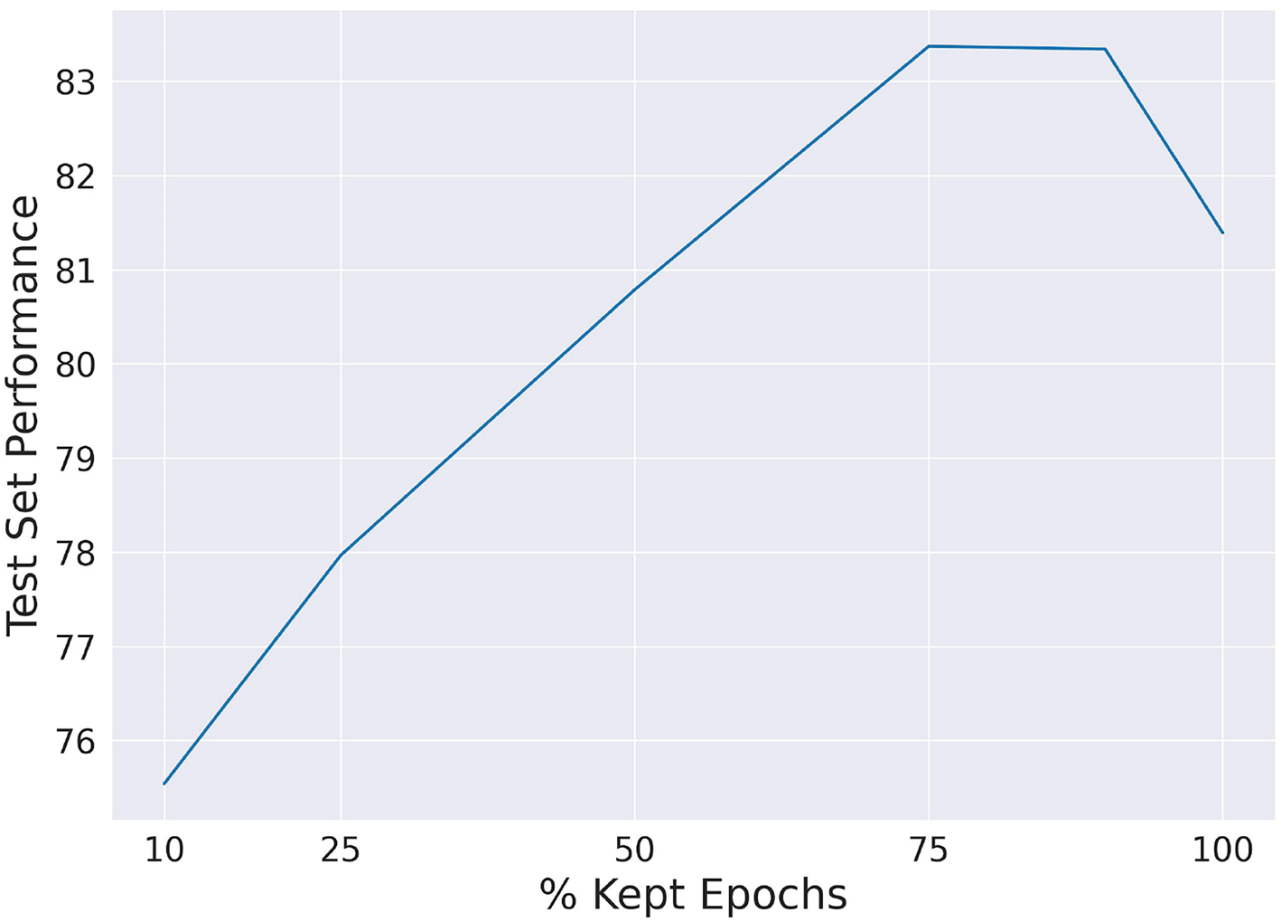
Test set performance as influenced by the exclusion of uncertain epochs. The figure shows the greatest performance improvements achieved across all ensembles and iterations

**Table 3:** Uncertainty - error decrease: Performance of various ensemble models using the proposed uncertainty approach

Metric	% kept epochs				
	90	75	50	25	10
Avg. Err. Decrease	3.5	5.4	6.6	5.5	2.9
Avg. Max Err. Decrease	5.5	9.5	13.6	16.5	16.3
Max Err. Decrease	9.9	21.4	32.0	31.9	24.0

The error decrease is calculated from the improvement in accuracy and is presented as the “percentage of error decrease”. The “Avg. Error Decrease” signifies the average error reduction across all ensemble models and iterations (folds). “Average Maximum” denotes the mean value of the largest error reductions observed across all ensembles. The “Maximum Error Decrease” refers to the highest error reduction observed across all ensemble models. This visualization allows for the quantification of the impact of the uncertainty method on performance

Figure 3 presents the best-achieved improvement across all ensembles, leading to an overall maximum of 32.0% decrease in prediction error. Table 3 presents the overall performance of the method with an average, average maximum, and maximum error decrease across all ensembles. Considering the average error decrease, we observed that the ensembles consistently improved, on average, as uncertain epochs were removed, up to a threshold of 50%. Uncertain epochs are based on variance metrics from the ensemble model predictions. These findings suggest that a reduction of uncertain epochs had a positive impact on the ensemble’s performance. However, beyond the 50% threshold, the performance of the ensembles began to decline (see Table 3).

## Discussion

In this study, we successfully repurposed two time-series models to predict sex directly from automatically cleaned EEG data, achieving high accuracy on a large test set. We examined deep ensemble configurations on the same problem to explore their robustness and potential performance enhancement. Our findings revealed high performance across all ensembles, with no significant differences observed compared to the already high accuracy of individual models. A data-driven exploration revealed that the decisive features for sex prediction were present across all frequency bands. However, we found that the models’ capability to identify these features was more consistent in lower frequencies. Finally, we employed an uncertainty approach to discard uncertain epochs based on a straightforward variance metric. This strategy led to an average error reduction across all ensembles and folds.

### Single model performance

The results obtained from our study affirm the capability of DL models to discover complex sex-specific patterns with a high degree of accuracy in EEG data. Notably, the high accuracy provides a compelling basis to believe these models can be further fine-tuned and applied to detect and understand more complex neurological and cognitive states. While the direct clinical utility of sex prediction may be limited, understanding sex-specific differences in EEG patterns has broader implications. Sex is a significant factor in many neurological diseases, and accounting for these differences can enhance the accuracy of diagnoses and contribute to the development of personalized treatments. Incorporating sex as a critical variable in disease research could help improve model generalization and diagnostic performance.

### Sex prediction using deep learning

In the present study, InceptionNetwork and EEGNet outperformed earlier research in sex prediction directly from EEG data. On a larger test set of 356 subjects, these models achieved a mean accuracy of 90.7% (± 2.4) and 90.5% (± 1.6) on per-subject prediction, respectively. In per-epoch prediction InceptionNetwork achieved a mean accuracy of 88.0 (± 1.6) and AUC of 0.947, and EEGNet a mean accuracy of 88.3 (± 1.5) and AUC of 0.938. Comparison of performance showed non-overlapping 95% confidence intervals, typically implying statistical significance. This suggests a significant improvement, although variations in dataset size may account for the performance increase.

Van Putten et al. [26] first introduced the concept of predicting sex directly from EEG using DL, applying it on a sizable test set of 308 subjects (1000 for training and validation), attaining an 81% accuracy for per-subject classification. They highlighted the role of sex predictions in the beta band of EEG. Subsequently, Bučková et al. [27] confirmed van Putton’s et al. [26] approach on a separate dataset of 144 subjects, achieving 83.45% (leave-one-subject-out scheme). Truong et al. [28] later demonstrated a high mean accuracy of 87% (± 0.4) per-subject and per-epoch 83.1% (± 0.3) on the same Child Mind Institute dataset as used in our study, but with a smaller test set of 197 subjects. Their cleaning pipeline was possibly more liberal as they only had a final perfectly balanced dataset of 1574 vs. our 1780, or they used an earlier version containing fewer subjects. In addition, they employed a larger 30% validation set at the cost of a smaller test set of 10%. We prioritized a larger test set of 20% at the cost of a smaller validation set of 20%. Consequently, our training set included more subjects than Truong et al. [28], while our validation set was comparatively smaller. Truong et al. [28] manually selected 24 channels that overlapped with those used by van Putten et al. [26]. However, we used all channels as it yielded good performance. Similarly to van Putten et al. [26] and Truong et al. [28], our work demonstrated that EEG time series may successfully be used as input to DL.

### Deep learning ensemble performance

The results, which were not statistically significant, did not meet our initial expectations of ensembles outperforming single models. Importantly, every single model in our study demonstrated high performance, which could account for the observed outcomes. The performance of the individual models may represent the practical limit for distinguishing between male and female EEG activity, as traditional brain research has reported overlapping EEG patterns between the sexes [29, 30]. However, factors such as the specific ensemble strategies employed might explain this performance convergence. Despite these results, the findings contribute valuable insights for future research and development efforts.

Our ADS ensembles encountered overfitting issues when we incorporated additional dense layers. Reducing these layers enhanced generalizability but limited the diversity of achievable architectures through dropout. Furthermore, adding additional layers led to an increased instability between runs relative to other ensemble types. Ensembles employing random weights (both uniform and normal distributions) showed strong performances, mirroring the results seen in depth and model ensembles. The lack of notable performance enhancement across these groups may be due to the substantial similarity among the models within each ensemble. To enhance ensemble performance, fostering greater dissimilarity among models could prove beneficial. Models with high similarity often converge to similar regions in the optimization landscape, potentially undermining the ensemble approach’s benefits. Prior research, such as Fawaz et al. [31], has shown superior results with ensembles comprising distinct model architectures for time series analysis. Although the performance of the ensemble models in this study did not significantly exceed that of single models and involved in some cases greater computational expense and complexity, the advantages of uncertainty modeling offered by ensembles should not be overlooked. The ability of ensembles to generate uncertainty estimates can enhance model performance, as shown in this work, and increase the reliability, trustworthiness, and applicability of future systems.

### Frequency explanation

Prior research by van Putten et al. [26] indicated that, among all frequency bands, the beta band (12–25Hz) was most important for sex prediction in a sample aged 18–98 years. Bučková [27] confirmed these findings in a separate dataset involving participants aged 18–65 with major depressive disorder. Jochmann et al. [29] reports that the EEG topograhy were critical in detecting the sex in their study, and that none of the frequencies in particular were important. In contrast, our results reveal that the ability to detect sex with high predictive value is evident across all frequency bands. Among the frequency bands investigated, delta, theta, and low-beta demonstrated higher consistency in our research. It is noteworthy that other studies solely identified this pattern in the low-beta (12–20Hz) band [26]. Our model demonstrated improved optimization across all iterations and subsets. However, it encountered challenges in the gamma, high-beta, and alpha bands. These deviations from prior findings may be attributable to our relatively young study sample (ages 5–21 years). Traditional EEG studies have reported sex differences in frequency bands, but research in this field is limited and results are conflicting [32]. Cave et al. [32] attempted to confirm and clarify these findings, reporting that “females had greater overall amplitudes in delta, alpha, and beta, enhanced midline activity in theta, and parietal and midline activity in the alpha and beta bands.” These conclusions both support and contradict multiple previous studies, highlighting inconsistencies across different frequency bands; see [32] for more information.

Several factors, including initial starting weights [14, 31], can be attributed to the *unstable* performance across the frequency bands. Interestingly, instability tended to occur in the higher frequency bands in our experiments. Higher frequency bands often capture fine-grained details and short-term variations in the data, suggesting that the initially selected weights significantly impact the model’s ability to effectively capture and utilize these details. The consistent filter size used across all experiments with various frequency bands could provide another explanation for our model’s performance instability. Larger filters favor slower frequencies due to their extended time window, which is more compatible with less frequent changes. However, these larger filters might struggle with higher frequencies, which change more rapidly, which may have affected the results, as InceptionNetwork’s filters might have favored slower frequencies. Nevertheless, the model’s performance in the low-beta range suggests that this explanation might not fully account for the observed behavior.

### Uncertainty

Our study underlines the potential to employ variance as a metric to discard uncertain samples in sex prediction using multiple different ensemble approaches. In the best scenario, this straightforward strategy achieved a 32% reduction in prediction error, particularly notable in the lowest performing ensemble type using frequency bands. This method demonstrated its effectiveness for boosting performance in underperforming ensembles, making it a highly beneficial strategy for significant performance improvements. Despite some runs showing performance degradation, the majority consistently exhibited performance improvement across all ensemble approaches. Previously, Fiorillo et al. [33] used a methodology similar to ours, employing variance (and, in their case, mean) to select certain epochs within the domain of sleep stage prediction using a single ensemble approach, MCD. Their observations of improved predictions through epoch selection is consistent with our findings. Additionally, another study has suggested enhancing EEG predictions by incorporating uncertainty by utilizing MCD and the Bhattacharyya distance metric [25].

Our results demonstrated a positive trend in performance up to a certain threshold (50%), beyond which a decline occurred. This phenomenon may have multiple causes. Our methodology’s absence of a certainty threshold might have inadvertently led to discarding epochs where the model had a relatively high degree of certainty. Furthermore, diversifying our metrics to capture model uncertainty more effectively could be advantageous. For instance, incorporating measures like predictive entropy or mutual information could enhance prediction reliability. Additionally, considering aleatoric uncertainty, which Bayesian DL techniques can model, might prove advantageous to model uncertainty from various sources, such as noise in the measurement process, electrode placement variability, and inherent biological variations. Increased noise may have caused models to concur on certain predictions, indicating low variance, even when those predictions were incorrect. While this study did not compare the uncertainty of different models, the implemented uncertainty framework reduced errors. This simple approach exhibits significant potential for performance improvement. It could be instrumental in enhancing proficient models, specifically bolstering the robustness of systems using ensemble-based predictions. Suppose certain models in the ensemble struggle or show uncertainty over multiple samples/epochs from a subject or similar data structures. This strategy effectively identifies and excludes these uncertain elements, boosting the system’s robustness and overall performance. In an added layer of utility, the variance metric can be paired with prediction outputs which could provide users, particularly in a clinical context, with an indication of the model’s reliability. Pairing predictions with measures of certainty can aid clinicians in making crucial decisions. Identifying uncertain samples as potentially risky predictions underscores the importance of involving medical experts in collaborative decision-making with DL systems. Our findings not only improve prediction accuracy but also underscore the practical applicability of these models in real-world settings.

### Limitations

In this study, we focused exclusively on a single data-driven explainability technique applied to one model, specifically the InceptionNetwork. Future work should also include model-specific explainability methods to confirm the consistency of the features used by the introduced model with subjects within the same class. Similarly, we only used one metric for uncertainty, whereas including several metrics that capture both the aleatoric and epistemic uncertainty will be relevant for future work. We did not set a threshold for removing uncertain samples/epochs, possibly leading to excessive, blind data reduction. Physiological factors associated with the EEG could introduce classification bias. For instance, females tend to have thinner skulls than males [34], potentially leading to higher average EEG amplitudes in females.

Furthermore, the dataset includes children and adolescents representing a broad spectrum of developmental clinical characteristics. Without linking subjects’ diagnostic information to their labels, potential confounding factors from neurological and psychiatric pathologies could impact male and female differentiation. Despite these challenges, our models robustly and accurately predicted sex from pure EEG signals.

Lastly, our methods have been validated on a single dataset, and future studies would benefit from extending this validation, particularly regarding the frequency aspects, across datasets that include different age groups and demographics.

## Conclusion

In this study, we achieved notable performance in predicting sex from raw EEG time series using well-established EEG and time-series models. Our data-driven approach revealed determinative sex-related features across all frequency bands. While DL-based ensemble methods did not yield a significant improvement over single models, the approach facilitated the integration of uncertainty modeling, contributing to improved prediction performance. The results hold promising potential for various EEG applications and offer exciting prospects for levering similar approaches in other data-rich domains. Future work involves to further explore the quantification of uncertainty, in particular new metrics and Bayesian DL.

## Data Availability

The datset, preprocessing and experiment code is available. Dataset, see paper [16]. [Downloaded: Dec. 2022]. The dataset can be downloaded automatically using this script that is available here. The automatic preprocessing pipeline is supplied here. All code for all experiments, models and method is here.

## Abbreviations

EEG: Electroencephalography
DL: Deep learning
AI: Artificial intelligence
ML: Machine learning
AUC: Area under the curve
MCD: Monte Carlo dropout
ADS: Adapted dropout strategy
